# Leaf size variations in a dominant desert shrub, *Reaumuria soongarica*, adapted to heterogeneous environments

**DOI:** 10.1002/ece3.6668

**Published:** 2020-08-19

**Authors:** Xingke Fan, Xia Yan, Chaoju Qian, Daoura Goudia Bachir, Xiaoyue Yin, Peipei Sun, Xiao‐Fei Ma

**Affiliations:** ^1^ Key Laboratory of Stress Physiology and Ecology in Cold and Arid Regions, Gansu Province Department of Ecology and Agriculture Research Northwest Institute of Eco‐Environment and Resources Chinese Academy of Sciences Lanzhou China; ^2^ University of Chinese Academy of Sciences Beijing China; ^3^ School of Life Sciences Nantong University Nantong China

**Keywords:** arid Central Asia, common garden, environmental change, leaf size, local adaptation, *Reaumuria soongarica*

## Abstract

The climate in arid Central Asia (ACA) has changed rapidly in recent decades, but the ecological consequences of this are far from clear. To predict the impacts of climate change on ecosystem functioning, greater attention should be given to the relationships between leaf functional traits and environmental heterogeneity. As a dominant constructive shrub widely distributed in ACA, *Reaumuria soongarica* provided us with an ideal model to understand how leaf functional traits of desert ecosystems responded to the heterogeneous environments of ACA. Here, to determine the influences of genetic and ecological factors, we characterized species‐wide variations in leaf traits among 30 wild populations of *R. soongarica* and 16 populations grown in a common garden. We found that the leaf length, width, and leaf length to width ratio (L/W) of the northern lineage were significantly larger than those of other genetic lineages, and principal component analysis based on the in situ environmental factors distinguished the northern lineage from the other lineages studied. With increasing latitude, leaf length, width, and L/W in the wild populations increased significantly. Leaf length and L/W were negatively correlated with altitude, and first increased and then decreased with increasing mean annual temperature (MAT) and mean annual precipitation (MAP). Stepwise regression analyses further indicated that leaf length variation was mainly affected by latitude. However, leaf width was uncorrelated with altitude, MAT, or MAP. The common garden trial showed that leaf width variation among the eastern populations was caused by both local adaptation and phenotypic plasticity. Our findings suggest that *R. soongarica* preferentially changes leaf length to adjust leaf size to cope with environmental change. We also reveal phenotypic evidence for ecological speciation of *R. soongarica*. These results will help us better understand and predict the consequences of climate change for desert ecosystem functioning.

## Graphical abstract

The variations in leaf size along environmental gradients within the species range of Reaumuria soongarica were investigated. Our study shows that there are significant differentiations of leaf size and environmental conditions between genetic lineages in this species. Leaf width variations in heterogeneous environments are partly affected by local adaptation and this shrub preferentially changes leaf length to adjust leaf size to cope with environmental change.

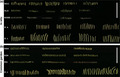

## 1 INTRODUCTION

As the main organ of photosynthesis, transpiration, and gas exchange, leaves deeply influence the growth, reproduction, and survival of plants, and even affect the energy flow and chemical cycling of most terrestrial ecosystems (Wright et al., 2004). Most leaf functional traits, such as morphological and physiological characteristics, vary considerably among and within species (Albert et al., 2010; Kattge et al., 2011). These variations are closely related to the environment, and the relationships between leaf traits and the environment are increasingly used to study the impacts of climate change on ecosystem functioning, especially in the tundra (Albert et al., 2010; Bjorkman et al., 2018; Myers‐Smith, Thomas, & Bjorkman, 2019; Soudzilovskaia et al., 2013). In such research, leaf size is one of the most commonly used functional traits because it strongly affects light interception and leaf temperature (Falster & Westoby, 2003; Gates, 1968) and has an important influence on leaf energy balance, water balance, and aboveground biomass accumulation (Farquhar, Buckley, & Miller, 2002; Parkhurst & Loucks, 1972; Street, Shaver, Williams, & Van Wijk, 2007; Wang et al., 2019; Wright et al., 2017). Also, leaf size varies greatly [from 0.79 mm^2^ (*Wolffia arrhiza* and *Azolla microphylla*) to 2.79 m^2^ (*Victoria amazonica*)] between species (Diaz et al., 2016) and has marked variations among and within species along environmental gradients (Hovenden & Vander Schoor, 2004; Wright et al., 2017). Therefore, studying the correlation between leaf size and the environment is important for understanding plant growth strategies and predicting the responses of plant populations, communities, and ecosystems to environmental change (Bjorkman et al., 2018; Byars, Papst, & Hoffmann, 2007; Guerin, Wen, & Lowe, 2012; Hudson, Henry, & Cornwell, 2011; Wright et al., 2017). Additionally, this type of research can also improve our understanding of local adaptations made by plants to particular environments (Alcantara, Bastida, & Rey, 2010; Byars et al., 2007; Gonzalo‐Turpin & Hazard, 2009; Steane et al., 2017).

As early as the 19th century, biogeographers noted that leaf size is generally larger at lower latitudes (Schimper, 1903). Since then, many environmental factors have been confirmed to strongly affect leaf size, such as mean annual precipitation (MAP), mean annual temperature (MAT), altitude, light exposure, wind speed, and soil fertility (Hovenden & Vander Schoor, 2004, 2006; McDonald, Fonseca, Overton, & Westoby, 2003; Peppe et al., 2011; Wu, Zhang, Zhang, Wang, & Yu, 2016). For instance, leaf size generally decreases with increasing altitude and decreasing MAP (Hovenden & Vander Schoor, 2004; Wright et al., 2017). However, the relationships between leaf size and environmental factors are not fixed and can vary in different regions or within different species. Thus, the key environmental factors influencing leaf size are still debatable. For example, Wright et al. (2017) noted that leaf size increases with increasing MAP at warmer sites on the global scale, but has no correlation with MAP at colder sites. Perhaps how leaves respond to environmental changes results from the combination of environmental factors they are exposed to (Wright et al., 2017), and differences in response strategies employed between species (McDonald et al., 2003). In fact, many environmental gradients (e.g., temperature, latitudinal, altitudinal) of leaf size can be explained by the leaf energy balance theory, which comprehensively considers the air temperature, radiation, and water condition (Gates, 1965; Leigh, Sevanto, Close, & Nicotra, 2017; Lusk et al., 2018; Parkhurst & Loucks, 1972; Wright et al., 2017). In short, the heat exchange between leaves and the surrounding air is slower in larger leaves due to thicker boundary layer effects, which may cause larger leaves to face a greater risk of serious heat damage in hot and arid environments, or frost damage on clear nights in cold regions (Leigh et al., 2017; Lusk et al., 2018; Parkhurst & Loucks, 1972; Wright et al., 2017).

In addition to environmental factors, the genetic factor also influences the variation in leaf size (Powell & Lenhard, 2012). The current relationships between leaf traits and environmental factors in some species may be the result of long‐term adaptation to different habitats. Thus, these traits change little in response to short‐term climate variation (Cordell, Goldstein, Mueller‐Dombois, Webb, & Vitousek, 1998; Zhu et al., 2012). For instance, the altitudinal variation in leaf size of *Metrosideros polymorpha* remained unchanged in a common garden experiment, suggesting that these changes were caused by local adaptation to particular altitudes (Cordell et al., 1998). Byars et al. (2007) used transplant experiments and a common garden experiment to show that the altitudinal trends of leaf length and plant circumference in *Poa hiemata* are affected by both genetic and environmental factors. Another study on *Nothofagus cunninghamii*, however, showed that the trend of leaf size along an altitudinal gradient in this species was mainly influenced by phenotypic plasticity (Hovenden & Vander Schoor, 2004). Somehow, the pattern of leaf size variation is not fixed within lineages (McDonald et al., 2003). Thus, to make more accurate use of the relationships between leaf size and environmental factors to predict the consequences of climate change, it is necessary to conduct a common garden experiment in advance. This will clarify which aspects of leaf size variation in a plant species are caused by local adaptation, and which are the result of phenotypic plasticity.

As one of the largest arid zones in the world, arid Central Asia (ACA) harbors diverse desert ecosystems, the function, and structure of which are particularly sensitive to climate change (Kong, Zhang, Singh, & Shi, 2017; Seddon, Macias‐Fauria, Long, Benz, & Willis, 2016; Yin, Hu, Chen, & Tiyip, 2016; Zang, Min, de Dios, Ma, & Sun, 2020). Zhu et al. (2019) have indicated that the responses of vegetation to climate change in desert areas of northern China differ regionally, and vegetation dominated by taller woody plants is more sensitive to climate change than that dominated by dwarf shrubs. Moreover, long‐term climate differences within ACA can not only change the plant traits and biomass of different populations, but also affect their reproductive strategies; for example, the northern genetic group of *Agriophyllum squarrosum* endemic to ACA has smaller vegetative organs and larger seeds than the southern group, due to the long‐term adaptation to different precipitations and wind speeds (Yin, Qian, Chen, Yan, & Ma, 2016; Yin, Zhao, et al., 2016). It should be noted that the rate of climate warming in ACA has been larger than the mean warming rate of the global land area in past decades (Hu, Zhang, Hu, & Tian, 2014), and MAPs in most areas of ACA have increased rapidly (Chen, Huang, Jin, Chen, & Wang, 2011). These changes might increase aridity in some areas, leading to a decline in productivity and even biodiversity (Bellard, Bertelsmeier, Leadley, Thuiller, & Courchamp, 2012; Zhu et al., 2019). To mitigate the impact of rapid climate change on desert ecosystems, it is vital that the development of management interventions, such as ecological restoration projects, and conservation policies, is founded on a comprehensive understanding of the ecological consequences of climate change in ACA (Malhi et al., 2020; Vogt et al., 2011; Zhu et al., 2019). However, little attention has been paid to understand the responses of plant traits within species to in situ environmental change in ACA, and so as in other arid regions.

*Reaumuria soongarica* (Pall.) Maxim, belonging to the family Tamaricaceae, is a typical xerophyte shrub widely distributed in ACA (Liu, Qiu, Pu, & Lu, 1982; Shi et al., 2013). It can grow in different habitats, including the Qinghai‐Tibet Plateau, the Loess Plateau, the Taklimakan Desert, and the Gurbantunggut Desert. As an important constructive and dominant species, *R. soongarica* plays a vital role in maintaining the stability of these fragile desert ecosystems (Liu et al., 1982; Ma, Chen, Qiang, & Wang, 2005) and thus can reflect the health of the desert communities in which it inhabits. With the continuous desertification in ACA (Guo et al., 2002), this tertiary relic shrub has evolved specific leaf traits to adapt to this harsh environment, such as terete shape, thick cuticles, and hollow stomata with low density (Liu et al., 1982, 2018). Moreover, through field observations, we found that leaf sizes vary greatly in different populations of this species. Recent experiments have shown that the aboveground biomass and the growth period of the seedlings of this shrub (both of which are closely related to leaf size) are changed by short‐term variations in rainfall quantity and interval, respectively (Zhang, Shan, & Li, 2018). Furthermore, the processes of aridification in ACA further led to the differentiation of *R. soongarica* into three distinct genetic lineages (Li et al., 2012; Shi et al., 2020; Yin et al., 2015). Thus, *R. soongarica* provides an excellent opportunity to study how leaf traits of a widespread desert species respond or adapt to heterogeneous environments across ACA.

In this study, we collected leaf size data of both in situ and common garden populations of *R. soongarica* in northwest China to investigate how environmental and genetic differences affect leaf size variations in this species. Theoretically, intraspecific trait variation is affected by trade‐offs between abiotic stress and biotic factors (such as species richness and trait associations) (Agrawal, 2020; Kuppler et al., 2020). Considering the sparse vegetation and limited resources in ACA, we hypothesized that leaf size variations of this constructive shrub were more impacted by abiotic stress gradients. On the other hand, as *R. soongarica* had evolved into three genetic lineages with their own ecological niches, these phenotypic variations could also be shaped by the genetic structure. To elucidate the above hypotheses, we aimed to (a) investigate the variations in leaf size of *R. soongarica* under different environments and different lineages; (b) reveal whether the relationships between leaf size and the major environmental factors in this extreme environment are consistent with the general rules; and (c) address whether phenotypic variations in leaf size are influenced by local adaptation or plasticity based on a common garden experiment. Using space‐for‐time substitution, these spatial trait‐environment relationships will help us better understand and predict the consequences of climate change on the leaf sizes of desert plants and even the functioning of desert ecosystems.

## 2 MATERIALS AND METHODS

### 2.1 Sample collection of the wild populations

**FIGURE 1:**
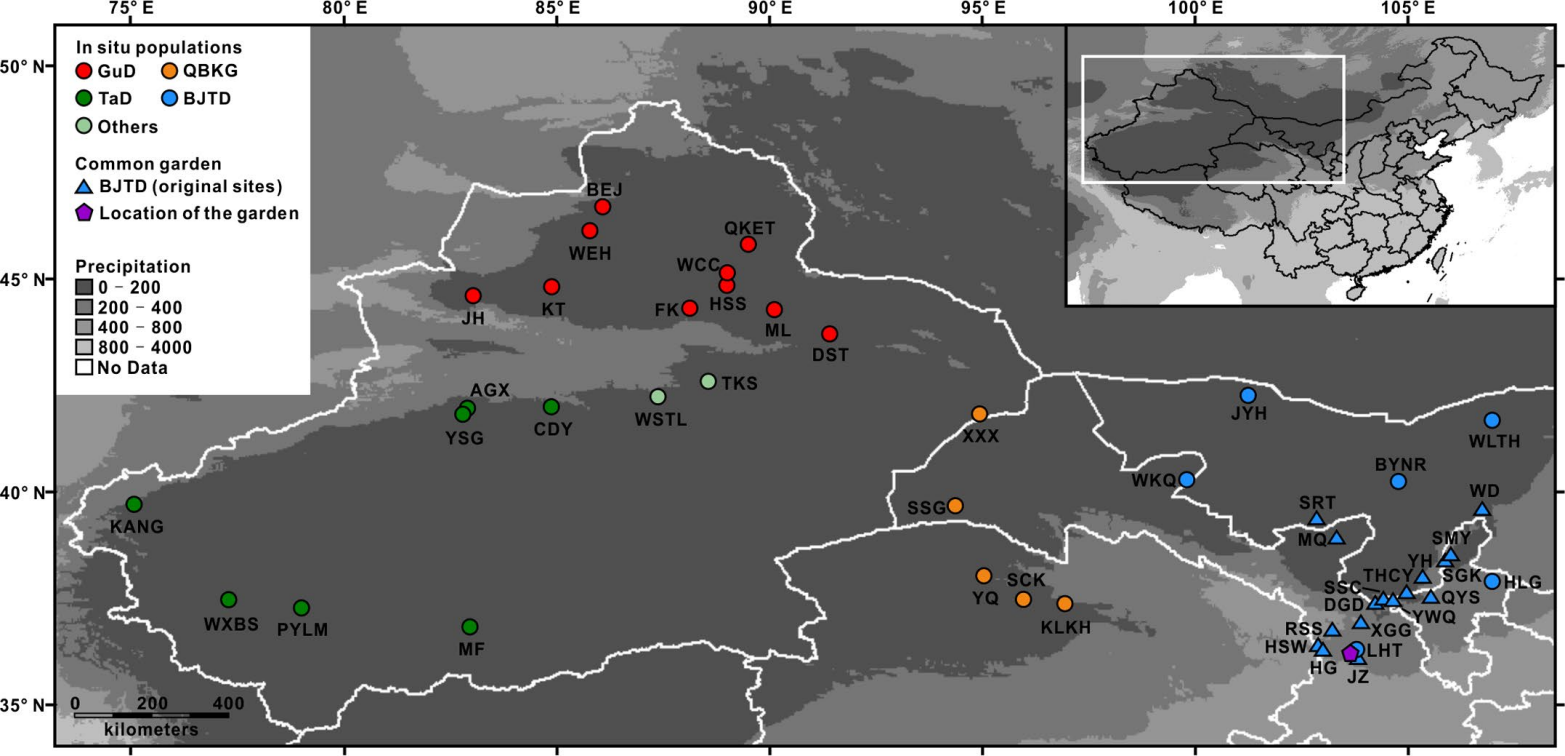
Geographic locations of the *Reaumuria soongarica* populations used in this study. The blue triangles represent the original site of populations grown in the common garden. The four main genetic groups of *R. soongarica* are indicated by different colors. The purple polygon represents the location of the common garden. Precipitation is measured in millimeters

During the summer of 2012 and 2013, a total of 287 *R. soongarica* individuals were sampled in situ from 30 wild populations (Table 1). These sampling sites covered the whole range of *R. soongarica* in northwest China (Figure 1). In each population, four to ten healthy shrubs that had not been eaten by animals were randomly selected. To reduce the sampling bias, all individuals sampled in each population were spaced more than 30 m apart. The middle section of three leafy branches located in the upper part of each sample was then harvested and dried by silica gel. Additionally, there are two other *Reaumuria* species, *R. trigyna* and *R. kaschgarica,* also found in northwest China (Hao, Zhang, Wang, & Zhang, 2014). Fresh leaves of ten individuals of *R. trigyna* and *R. kaschgarica* were also sampled in the wild. Tissue samples were kept at 4°C until measurement.

**TABLE 1:** Geographic locations and climate data of the in situ populations and common garden populations of *Reaumuria soongarica*

Population code	Location	Longitude (°E)	Latitude (°N)	Altitude (m)	Group	Lineage	Sample size	MAT (°C)	MAP (mm)
WLTH	Wulatehouqi, IM	107.005	41.700	1,458	BJTD	Eastern	10	5.10	132
HLG	Hongliugou, NX	107.000	37.902	1,442	BJTD	Eastern	10	7.70	274
BYNR	Bayannuori, IM	104.766	40.254	1,312	BJTD	Eastern	10	7.43	93
LHT	Laohutai, GS	103.821	36.269	1,853	BJTD	Eastern	10	7.87	330
JYH	Juyanhai, IM	101.244	42.251	903	BJTD	Eastern	10	8.68	34
WKQ	Weikengquan, GS	99.819	40.279	1,159	BJTD	Eastern	10	8.83	69
KLKH	Keluke Lake, QH	96.967	37.346	2,946	QBKG	Eastern	10	3.72	148
SCK	Sanchakou, QH	95.973	37.456	3,289	QBKG	Eastern	10	1.46	115
YQ	Yuqia, QH	95.029	38.028	3,221	QBKG	Eastern	10	1.14	69
XXX	Xingxingxia, XJ	94.939	41.824	1,596	QBKG	Eastern	9	6.06	61
SSG	Shashangou, GS	94.367	39.664	1,670	QBKG	Eastern	10	7.68	32
DST	Dashitou, XJ	91.437	43.699	1,638	GuD	Northern	10	4.51	106
ML	Mori, XJ	90.121	44.271	731	GuD	Northern	10	6.76	121
QKET	Qiakuertu, XJ	89.514	45.816	1,029	GuD	Northern	10	3.73	178
WCC	Wucaicheng, XJ	88.993	45.158	773	GuD	Northern	10	5.47	176
HSS	Huoshaoshan, XJ	88.989	44.861	478	GuD	Northern	10	7.16	155
FK	Fukang, XJ	88.125	44.315	491	GuD	Northern	10	8.13	170
BEJ	Buerjin, XJ	86.090	46.688	1,044	GuD	Northern	10	4.75	144
WEH	Wuerhe, XJ	85.780	46.127	329	GuD	Northern	4	8.43	114
KT	Kuitun, XJ	84.861	44.797	313	GuD	Northern	10	9.62	127
JH	Jinghe, XJ	83.019	44.619	420	GuD	Northern	10	7.62	118
CDY	Cedaya, XJ	84.860	42.007	1,078	TaD	Western	10	9.52	80
MF	Mingfeng, XJ	82.966	36.835	1,848	TaD	Western	10	9.25	37
AGX	Agexiang, XJ	82.892	41.946	1,543	TaD	Western	10	7.41	115
YSG	Yanshuigou, XJ	82.820	41.857	1,410	TaD	Western	7	8.60	102
PYLM	Piyalema, XJ	78.994	37.248	1,532	TaD	Western	10	11.98	38
WXBS	Wuxiabashi, XJ	77.276	37.478	1,841	TaD	Western	10	10.24	37
KANG	Kangsu, XJ	75.074	39.703	2,215	TaD	Western	10	6.09	170
TKS	Tuokexun, XJ	88.563	42.606	533	—	—	7	10.97	57
WSTL	Wusitala, XJ	87.396	42.227	1,256	—	—	10	8.59	108
DGD^a^	Dageda, GS	104.225	37.387	1,681	BJTD	Eastern	6	7.97	205
HG^a^	Honggu, GS	103.025	36.256	1,775	BJTD	Eastern	5	8.00	369
HSW^a^	Haishiwan, GS	102.883	36.350	1,920	BJTD	Eastern	5	7.27	377
MQ^a^	Minqin, GS	103.334	38.878	1,331	BJTD	Eastern	5	8.15	105
XGG^a^	Xiaogangou, GS	103.892	36.906	2,180	BJTD	Eastern	5	5.68	299
RSS^a^	Renshoushan, GS	103.246	36.731	2,193	BJTD	Eastern	6	5.65	356
JZ^a^	Jiuzhou, GS	103.822	36.092	1,675	BJTD	Eastern	6	9.02	337
QYS^a^	Quanyanshan, NX	105.561	37.489	1,202	BJTD	Eastern	5	9.53	216
SSC^a^	Shashichang, NX	104.433	37.464	1,695	BJTD	Eastern	3	7.78	205
YWQ^a^	Yiwanquan, NX	104.629	37.428	1,700	BJTD	Eastern	5	7.74	210
SRT^a^	Suoritaisumu, IM	102.849	39.327	1,277	BJTD	Eastern	6	8.18	87
THCY^a^	Tonghucaoyuan, IM	104.979	37.598	1,285	BJTD	Eastern	5	9.50	189
WD^a^	Wuda, IM	106.769	39.539	1,181	BJTD	Eastern	5	8.20	171
YH^a^	Yanhu, IM	105.347	37.950	1,348	BJTD	Eastern	7	8.76	198
SGK^a^	Sanguankou, NX	105.888	38.365	1,357	BJTD	Eastern	5	8.24	208
SMY^a^	Simaying, NX	106.011	38.502	1,175	BJTD	Eastern	5	8.88	193
*R. trigyna* ^a^	Wuda, IM	106.769	39.539	1,181	—	—	7	—	—

Abbreviations: IM, Inner Mongolia; GS, Gansu; NX, Ningxia; QH, Qinghai; *R. trigyna*, *Reaumuria trigyna*; XJ, Xinjiang.

^a^ These populations were used in the common garden experiment, and the data shown here were the information of their original geographic locations.

### 2.2 Common garden experiment

A total of 17 populations spaced at least 100 km apart were included in the common garden trial of *R. soongarica* (seven populations in Gansu, five in Ningxia, four in Inner Mongolia and one in Sinkiang; Table 1). In each population, the seeds were harvested from 20 different individuals 30 m apart and then planted at the Gaolan Station of the Agricultural and Ecological Experiment, Northwest Institute of Eco‐Environment and Resources, CAS (36.2368 °N, 103.7981 °E, 1,800 m above sea level). This common garden is in the southern margin of *R. soongarica* distribution range and belongs to the semi‐arid zone. The mean annual temperature is 8.15°C with a mean annual precipitation of 332 mm. In the spring of 2012, three seeds from each plant were first sown into a 10 cm diameter pot that contained a 1:1 mixture of loess and nutrient soil. After growing in the greenhouse for two months, the healthy seedlings were transplanted into the field. Finally, each population contained 10 to 15 seedlings, each of which came from one different female parent. In addition, one population of *R. trigyna* was also grown in the same field. After planting, the experimental shrubs were neither watered nor fertilized. The common garden plants were sampled in July 2017, according to the method described above. In each population, three to seven well‐grown shrubs were sampled. Unfortunately, there were no healthy plants coming from the Fukang (FK) population, which was the only population coming from Sinkiang.

### 2.3 Climate data collection

The climate varies greatly in the distribution area of *R. soongarica*. To assess the relationships between leaf traits and environmental factors, the long‐term climate data of all study sites, including in situ populations and the populations used for the common garden experiment, were obtained using DIVA‐GIS version 7.5 (http://www.diva‐gis.org/). The environmental layer with a 2.5 arc‐minute resolution for the present (Hijmans, Cameron, Parra, Jones, & Jarvis, 2005) was downloaded from the WorldClim database (http://www.worldclim.org/). These climate data included 19 bioclimatic variables, such as MAT and MAP (Table 2). The value of each variable is the mean of the observation data over 30 years (1960–1990).

**TABLE 2:** Bioclimatic variables and altitude of the different genetic groups of *Reaumuria soongarica*

Bioclimatic variable	Annotation	BJTD	QBKG	GuD	TaD	TaD	
						Southern edge	Northern edge
Bio1 (°C)^a^	Annual mean temperature	7.916 ± 1.165^a^	4.010 ± 2.851^b^	6.618 ± 1.928^ab^	9.011 ± 1.912^a^	10.489 ± 1.386	7.902 ± 1.486
Bio2 (°C)	Mean diurnal range	13.644 ± 0.711^b^	15.043 ± 0.736^a^	12.671 ± 0.894^c^	13.548 ± 1.289^b^	14.189 ± 1.508	13.067 ± 1.042
Bio3	Isothermality (Bio2/Bio7) (* 100)	30.975 ± 1.654^b^	34.159 ± 1.484^a^	25.034 ± 1.999^c^	30.107 ± 2.510^b^	32.077 ± 2.399	28.629 ± 1.403
Bio4	Temperature seasonality (STD * 100)	1,113.636 ± 114.927^b^	1,068.945 ± 90.489^b^	1,424.320 ± 92.803^a^	1,164.013 ± 66.212^b^	1,112.254 ± 17.143	1,202.833 ± 62.320
Bio5 (°C)^a^	Max temperature of warmest month	28.695 ± 2.351^ab^	24.860 ± 4.247^b^	30.920 ± 2.880^a^	30.071 ± 1.852^ab^	31.467 ± 0.737	29.025 ± 1.758
Bio6 (°C)	Min temperature of coldest month	−15.464 ± 1.421^a^	−19.220 ± 2.123^b^	−19.790 ± 1.922^b^	−14.914 ± 2.516^a^	−12.700 ± 2.007	−16.575 ± 1.181
Bio7 (°C)	Temperature annual range (Bio5‐Bio6)	44.159 ± 3.157^b^	44.080 ± 2.306^b^	50.710 ± 2.589^a^	44.986 ± 1.683^b^	44.167 ± 1.361	45.600 ± 1.804
Bio8 (°C)	Mean temperature of wettest quarter	19.851 ± 2.191^b^	16.690 ± 3.726^c^	21.742 ± 2.458^a^	21.021 ± 2.194^ab^	21.444 ± 1.269	20.704 ± 2.871
Bio9 (°C)^a^	Mean temperature of driest quarter	−6.399 ± 1.592^a^	−4.863 ± 6.967^ab^	−10.655 ± 2.040^b^	−3.790 ± 4.718^a^	0.900 ± 2.307	−7.308 ± 1.574
Bio10 (°C)^a^	Mean temperature of warmest quarter	20.974 ± 1.916^ab^	16.690 ± 3.726^b^	22.838 ± 2.595^a^	21.986 ± 1.850^ab^	23.050 ± 1.126	21.188 ± 2.004
Bio11 (°C)	Mean temperature of coldest quarter	−6.640 ± 1.608^a^	−9.703 ± 1.765^b^	−12.183 ± 1.238^c^	−6.671 ± 2.363^a^	−4.378 ± 1.419	−8.392 ± 0.789
Bio12 (mm)^a^	Annual precipitation	211.682 ± 101.475^a^	85.000 ± 46.125^b^	140.900 ± 27.323^ab^	82.714 ± 50.364^b^	37.333 ± 0.577	116.750 ± 38.326
Bio13 (mm)^a^	Precipitation of wettest month	55.727 ± 23.513^a^	21.000 ± 9.028^b^	22.500 ± 4.503^b^	16.714 ± 7.740^b^	9.000 ± 1.000	22.500 ± 3.873
Bio14 (mm)^a^	Precipitation of driest month	1.000 ± 0.617^b^	1.000 ± 0.707^b^	4.100 ± 1.663^a^	1.286 ± 1.496^b^	0.000 ± 0.000	2.250 ± 1.258
Bio15	Precipitation seasonality (CV)	103.390 ± 8.904^a^	106.134 ± 7.318^a^	51.573 ± 17.485^c^	84.286 ± 14.976^b^	86.010 ± 17.084	82.992 ± 15.773
Bio16 (mm)^a^	Precipitation of wettest quarter	128.091 ± 57.887^a^	53.800 ± 26.864^ab^	58.100 ± 8.465^b^	43.714 ± 22.559^b^	20.667 ± 3.055	61.000 ± 9.055
Bio17 (mm)^a^	Precipitation of driest quarter	4.364 ± 2.479^b^	4.000 ± 2.000^b^	15.900 ± 5.607^a^	5.000 ± 4.000^b^	2.000 ± 1.000	7.250 ± 3.948
Bio18 (mm)^a^	Precipitation of warmest quarter	120.727 ± 52.537^a^	53.800 ± 26.864^ab^	56.100 ± 9.445^b^	41.857 ± 24.836^b^	16.333 ± 4.163	61.000 ± 9.055
Bio19 (mm)^a^	Precipitation of coldest quarter	4.364 ± 2.479^b^	4.400 ± 2.191^b^	16.100 ± 5.322^a^	6.143 ± 4.140^b^	4.000 ± 1.732	7.750 ± 4.924
Altitude (m)^a^		1,504.636 ± 340.702^a^	2,544.400 ± 842.250^a^	724.600 ± 417.058^b^	1,638.143 ± 366.079^a^	1,740.333 ± 180.456	1,561.500 ± 477.538

^a^ These variables of different genetic groups were abnormally distributed, and the others were normally distributed. Data are means ± *SD* of the populations in each genetic group. In each row, mean with different lowercase letters (*p* < .05) are significantly different. The southern edge of TaD includes PYLM, WXBS, MF populations, and the rest populations of TaD belong to the northern edge.

### 2.4 Measurement of leaf traits

Leaves of *Reaumuria* plants are small, sessile, terete, and fleshy, so only leaf length and leaf width (the widest part of the leaf), which are closely related to leaf size, can be readily quantified. Moreover, leaf width is the main determinant of leaf boundary layer thickness, which affects the rate of heat exchange between leaves and the surrounding air (Leigh et al., 2017; Parkhurst & Loucks, 1972). Furthermore, like the needles of conifers, the irregular terete leaf shape of *R. soongarica* makes it difficult to measure leaf area accurately. Although several methods for measuring the total needle area have been reported, the direct measurement methods are time‐consuming, and the computational methods applied to some specific geometric figures are not quite accurate (Berninger & Nikinmaa, 1994; Brand, 1987; Davies & Benecke, 1980; Johnson, 1984; Sellin, 2000). Thus, in this study, we used leaf length and leaf width instead of leaf area to represent the leaf size of *R. soongarica*.

**FIGURE 2:**
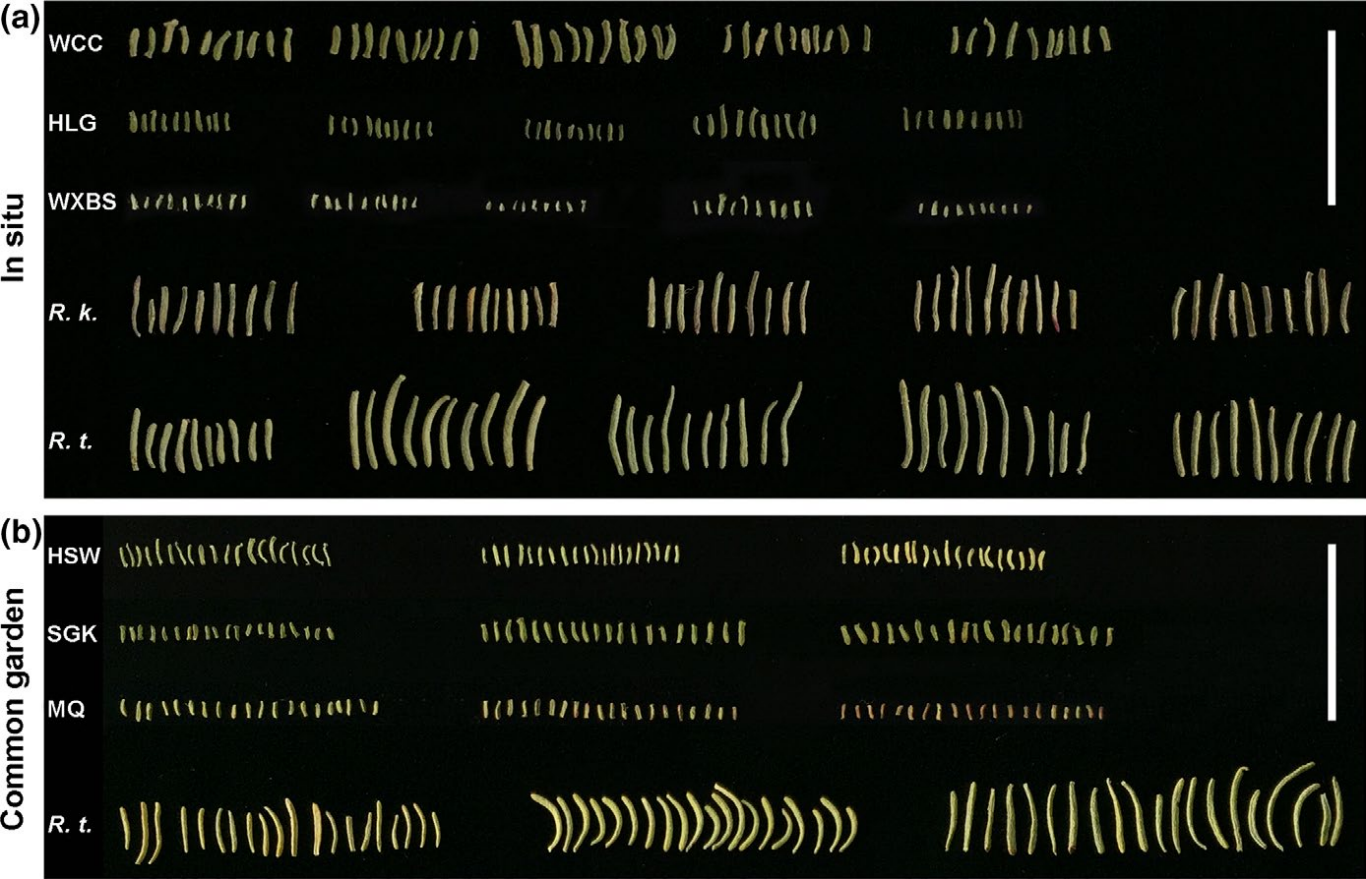
Leaves of the populations of *Reaumuria soongarica, R. kaschgarica,* and *R. trigyna*. (a) In situ populations, bar = 2 cm. The WCC, HLG, and WXBS populations of *R. soongarica* belong to different genetic lineages. Every ten stacked leaves come from the same in situ individual. (b) Common garden populations, bar = 2 cm. Twenty leaves were collected from each individual in the common garden. *R. k.*, *R. kaschgarica*; *R. t.*, *R. trigyna*

Several undamaged leaves were randomly selected, 10 for each wild individual and 20 per cultivated shrub. All the selected leaves were then placed on a black velvet background and digitally photographed (Figure 2). Measurements of leaf length and leaf width were acquired using the ImageJ image processing software (https://imagej.nih.gov/ij/; Schneider, Rasband, & Eliceiri, 2012). Subsequently, the leaf length to width ratio (L/W) of each leaf was calculated to describe leaf shape. The average of each trait in each population was calculated.

### 2.5 Data analysis

The Kolmogorov–Smirnov normality test was performed on all leaf morphological data and environmental data. Leaf length, leaf width, and L/W of some natural and common garden populations were abnormally distributed. Therefore, Kruskal–Wallis ANOVA tests were conducted to detect the differences in leaf characteristics among natural populations and among cultivated populations. According to previous studies (Shi et al., 2020; Yin et al., 2015), the populations used in this study were divided into different genetic groups and lineages (the eastern lineage contains the BJTD and QBKG groups, the western lineage contains the TaD group, and the northern lineage contains the GuD group). The differences in leaf traits of in situ samples between genetic lineages were also analyzed. Furthermore, we compared the leaf traits between garden populations and the in situ populations from the BJTD group (the original group of garden populations).

To detect correlations between leaf traits and environmental factors, linear regression analyses were conducted. Previous studies have shown that leaf traits can strongly correlate with many environmental factors (Hovenden & Vander Schoor, 2004, 2006; McDonald et al., 2003; Peppe et al., 2011; Wu et al., 2016). Meanwhile, some environmental factors are strongly correlated with each other at least within the distribution range of *R. soongarica* (Yin et al., 2015). Therefore, the effect of the environment on each leaf trait in the wild populations was further investigated using stepwise regression analyses to identify those environmental factors with the greatest influence. In these analyses, each leaf characteristic was taken in turn as a dependent variable, and every environmental factor (three geographic variables and 19 bioclimatic variables) was treated as an independent variable.

To analyze environmental heterogeneity among all sites used in this study, principal component analysis (PCA) was used to transform the environmental data, including altitude and 19 bioclimatic variables. Referring to the method of Lira‐Noriega and Manthey (2014), we used the first four principal components as coordinates to calculate the Euclidean distance between populations to represent their environmental distance. A significant correlation was observed between environmental distance and geographic distance between populations (Figure 9a). Therefore, we only executed partial Mantel tests to investigate the relationships between the phenotypic distance of leaf traits and environmental distance with 999 permutations. Additionally, the differences in environmental factors among different genetic groups were analyzed by one‐way ANOVA (variables with normal distributions) or Kruskal–Wallis ANOVA (variables with abnormal distributions).

The statistical analyses mentioned above were performed using SPSS 19.0 software (SPSS Inc., Chicago, IL, USA), GraphPad Prism 6.0 (GraphPad Software, Inc., San Diego, CA), and R 3.4.3 (R Development Core Team, 2017).

## 3 RESULTS

### 3.1 Leaf traits of in situ samples

**FIGURE 3:**
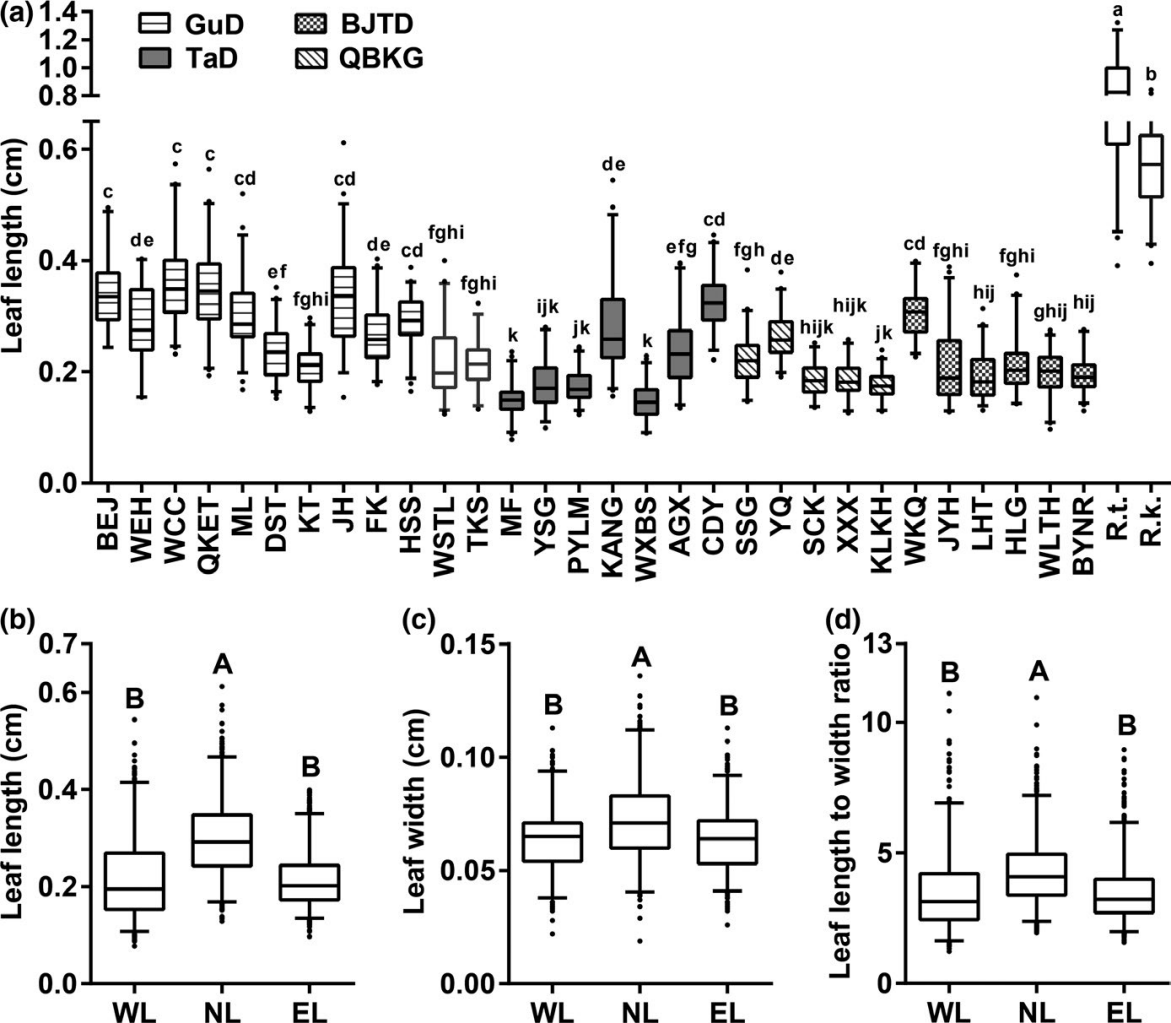
Variations in leaf traits among different populations (a) and different genetic lineages (b–d) of in situ *Reaumuria soongarica*. Each box plot shows the median, 95% confidence interval, and black dots as outliers. Different letters above each box indicate that there are significant differences in leaf traits among populations (lowercase, *p* < .05) and among lineages (uppercase, *p* < .01). *R. k.*, *R. kaschgarica*; *R. t.*, *R. trigyna*; WL, western lineage; NL, northern lineage; EL, eastern lineage

Leaves collected from some wild populations are shown in Figure 2a. Leaf length, leaf width, and L/W were all significantly different between the 30 in situ populations of *R. soongarica* (*p* < .001; Figure 3a; Table 3). The leaf length of the WCC population in northern Sinkiang was the longest (0.355 ± 0.067 cm), whereas the smallest leaf length (0.148 ± 0.032 cm) was found in the WXBS population from southern Sinkiang (Table 3). The coefficient of variation (CV) of leaf length within each in situ population varied considerably, ranging from 0.126 (KLKH population) to 0.305 (JYH population). However, none of the *R. soongarica* populations had leaves longer than either *R. trigyna* (0.82 ± 0.238 cm) or *R. kaschgarica* (0.581 ± 0.088 cm). The leaf width of *R. soongarica* populations ranged from 0.052 ± 0.009 cm (HLG population) to 0.085 ± 0.015 cm (WEH population), with some populations exhibiting leaf widths close to those of *R. trigyna* (0.089 ± 0.018 cm) and *R. kaschgarica* (0.077 ± 0.011 cm). The L/W of *R. soongarica* populations ranged from 2.385 ± 0.624 (MF population) to 5.217 ± 1.161 (CDY population), which was much lower than those of the other two species (Table 3).

**TABLE 3:** Statistics of measured leaf traits for the in situ *Reaumuria soongarica*

Lineage	Group	Population code	Leaf length (cm)		Leaf width (cm)		Leaf length to width ratio	
			Mean ± *SD*	CV	Mean ± *SD*	CV	Mean ± *SD*	CV
Northern	GuD		0.298 ± 0.076	0.257	0.072 ± 0.017	0.240	4.260 ± 1.223	0.287
		HSS	0.292 ± 0.044^cd^	0.150	0.075 ± 0.016^bc^	0.212	4.041 ± 0.902^def^	0.223
		FK	0.269 ± 0.055^de^	0.204	0.057 ± 0.012^gh^	0.203	4.899 ± 1.258^cd^	0.257
		ML	0.304 ± 0.060^cd^	0.199	0.073 ± 0.016^bcd^	0.219	4.299 ± 1.068^cdef^	0.248
		DST	0.236 ± 0.047^ef^	0.198	0.076 ± 0.012^bc^	0.161	3.189 ± 0.840^hij^	0.263
		BEJ	0.342 ± 0.058^c^	0.169	0.082 ± 0.017^ab^	0.211	4.318 ± 0.960^cdef^	0.222
		KT	0.209 ± 0.037^fghi^	0.179	0.057 ± 0.013^gh^	0.230	3.849 ± 1.271^efgh^	0.330
		WCC	0.355 ± 0.067^c^	0.189	0.084 ± 0.016^ab^	0.189	4.355 ± 1.091^cdef^	0.251
		QKET	0.346 ± 0.072^c^	0.208	0.079 ± 0.015^ab^	0.194	4.540 ± 1.163^cde^	0.256
		WEH	0.282 ± 0.061^de^	0.217	0.085 ± 0.015^ab^	0.178	3.366 ± 0.692^ghi^	0.205
		JH	0.335 ± 0.084^cd^	0.250	0.065 ± 0.013^defg^	0.197	5.206 ± 1.248^c^	0.240
Western	TaD		0.216 ± 0.082	0.379	0.064 ± 0.014	0.218	3.496 ± 1.444	0.413
		CDY	0.327 ± 0.047^cd^	0.145	0.065 ± 0.013^defg^	0.197	5.217 ± 1.161^bc^	0.222
		KANG	0.283 ± 0.082^de^	0.288	0.072 ± 0.014^bcde^	0.190	4.048 ± 1.441^efg^	0.356
		AGX	0.236 ± 0.057^efg^	0.242	0.058 ± 0.013^gh^	0.220	4.257 ± 1.502^def^	0.353
		YSG	0.179 ± 0.041^ijk^	0.227	0.064 ± 0.013^defg^	0.199	2.877 ± 0.774^ijk^	0.269
		PYLM	0.174 ± 0.028^jk^	0.158	0.069 ± 0.012^cdef^	0.177	2.620 ± 0.650^jk^	0.248
		MF	0.150 ± 0.029^k^	0.193	0.065 ± 0.014^defg^	0.213	2.385 ± 0.624^k^	0.262
		WXBS	0.148 ± 0.032^k^	0.218	0.053 ± 0.009^h^	0.175	2.879 ± 0.783^ijk^	0.272
Eastern			0.213 ± 0.055	0.257	0.064 ± 0.014	0.212	3.463 ± 1.083	0.313
	BJTD		0.219 ± 0.060	0.275	0.059 ± 0.011	0.191	3.822 ± 1.167	0.305
		WKQ	0.306 ± 0.042^cd^	0.138	0.063 ± 0.011^fg^	0.176	4.981 ± 1.077^c^	0.216
		BYNR	0.195 ± 0.029^hij^	0.147	0.063 ± 0.011^fg^	0.178	3.149 ± 0.555^hij^	0.176
		WLTH	0.196 ± 0.038^ghij^	0.195	0.058 ± 0.011^gh^	0.196	3.462 ± 0.910^ghi^	0.263
		LHT	0.192 ± 0.040^hij^	0.210	0.053 ± 0.007^h^	0.127	3.655 ± 0.820^fgh^	0.224
		HLG	0.213 ± 0.049^fghi^	0.228	0.052 ± 0.009^h^	0.172	4.191 ± 1.173^def^	0.280
		JYH	0.210 ± 0.064^fghi^	0.305	0.062 ± 0.011^fg^	0.179	3.495 ± 1.294^ghi^	0.370
	QBKG		0.207 ± 0.047	0.226	0.070 ± 0.013	0.192	3.023 ± 0.768	0.254
		SCK	0.186 ± 0.029^hijk^	0.156	0.064 ± 0.011^defg^	0.175	2.987 ± 0.647^ijk^	0.217
		YQ	0.263 ± 0.038^de^	0.144	0.079 ± 0.014^ab^	0.173	3.401 ± 0.699^ghi^	0.205
		KLKH	0.175 ± 0.022^jk^	0.126	0.067 ± 0.011^cdef^	0.169	2.679 ± 0.575^jk^	0.215
		SSG	0.221 ± 0.044^fgh^	0.197	0.068 ± 0.012^cdef^	0.174	3.344 ± 0.901^ghi^	0.270
		XXX	0.187 ± 0.030^hijk^	0.162	0.073 ± 0.014^bcd^	0.191	2.668 ± 0.650^jk^	0.244
Others	Others	TKS	0.214 ± 0.040^fghi^	0.184	0.076 ± 0.014^abc^	0.184	2.897 ± 0.771^ijk^	0.266
		WSTL	0.219 ± 0.063^fghi^	0.288	0.064 ± 0.011^efg^	0.169	3.468 ± 0.978^ghi^	0.282
*R. trigyna* (Outgroup)			0.820 ± 0.238^a^	0.290	0.089 ± 0.018^a^	0.202	9.214 ± 2.349^a^	0.255
*R. kaschgarica* (Outgroup)			0.581 ± 0.088^b^	0.151	0.077 ± 0.011^ab^	0.144	7.669 ± 1.547^ab^	0.202

Note

In each column, the mean of populations with different lowercase letters (*p* < .05) are significantly different.

Abbreviations: *SD*, standard deviation; CV, coefficient of variation.

The mean values of leaf length, leaf width, and L/W of the northern lineage were all significantly larger than those of other lineages (*p* < .001), while there was no significant difference in these traits between the eastern and western lineages (Figure 3b–d). Compared to other lineages, the western lineage had a higher CV for leaf length and L/W, but a moderate CV for leaf width (Table 3).

### 3.2 Environmental heterogeneity analysis

**FIGURE 4:**
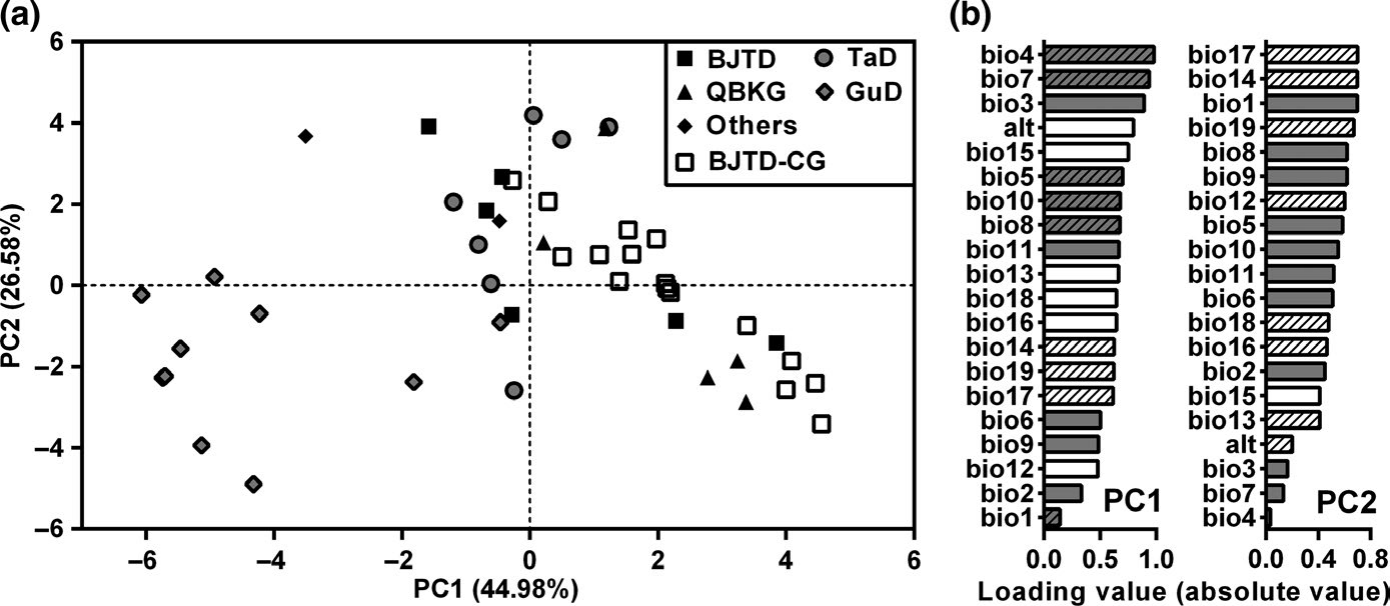
Principal component analysis of the environments of the 46 sampled sites of *Reaumuria soongarica* based on the altitude and 19 bioclimatic factors. (a) Environmental divergences along principal components 1 and 2. Each genetic group is highlighted by a different geometric figure. Others include TKS and WSTL populations. BJTD‐CG, the original sites of populations grown in the common garden belong to the BJTD group. (b) Ranked importance of variables based on their loading values in PC1 and PC2. The boxes with many diagonal lines represent negative loading values. The climatic factors related to temperature are indicated by filled boxes with gray, and the bioclimatic variables related to precipitation are highlighted by open boxes. Annotations of all bioclimatic factors are shown in Table 2

The climatic factors varied greatly across the 46 sites (30 in situ sites and the original sites of 16 garden populations) used in this study, with the MAT ranging from 1.14 to 11.98°C and MAP from 32 to 377 mm (Table 1). Moreover, elevations across these sites were very different, ranging from 313 to 3,289 m (Table 1). To analyze differences in the environments among these sites, altitude and 19 bioclimatic variables were used in PCA. The result showed that the environment of all populations from the GuD group (northern lineage), except for the DST population, was very different from that of other groups (Figure 4a). Although the sites from the BJTD, QBKG, and TaD groups were not clearly separated in the PCA result, some of them showed large environmental differences within each group. The first two PC axes explained 44.98% and 26.58% of the total variation of the environment, respectively (Figure 4a). In PC1, the top three loading variables were related to the variation of temperature: (a) temperature seasonality (bio4), (b) temperature annual range (bio7), and (c) isothermality (bio3) (Figure 4b). Meanwhile, three of the first four loading variables in PC2 were related to winter precipitation (Figure 4b). More specifically, there was strong seasonal variation in temperature and weak seasonal variation in precipitation in the GuD area, while the environment of the TaD group was hotter and drier, and the BJTD group had more precipitation with strong seasonality (Table 2). Additionally, the southern edge of the TaD region had higher MAT and a much lower MAP than the northern edge (Table 2).

### 3.3 Correlations between environmental factors and leaf traits in wild populations

**FIGURE 5:**
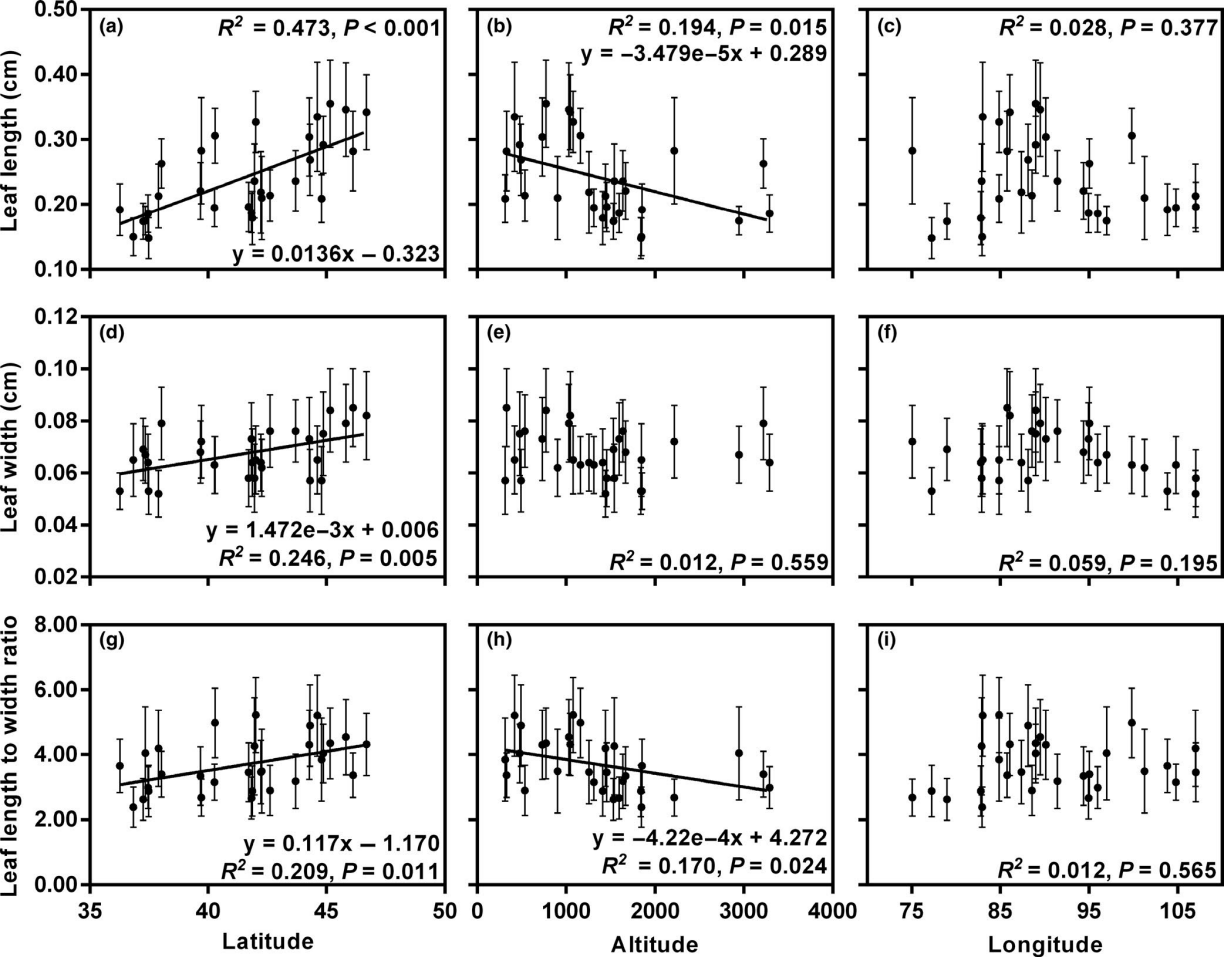
Relationships between leaf traits and geographic factors for the 30 in situ populations of *Reaumuria soongarica.* Each point is the mean of a trait in one population ± *SD*. Units: latitude (°N); altitude (m); longitude (°E)

The linear regression analyses revealed that the leaf length of in situ populations had a significant positive correlation with the latitude (*R^2^* = 0.473, *p* < .001; Figure 5a) and a significant negative correlation with the altitude (*R^2^* = 0.194, *p* = .015; Figure 5b). The leaf width and L/W also increased significantly with increasing latitude (*R^2^* = 0.246, *p* = .005 and *R^2^* = 0.209, *p* = .011, respectively; Figure 5d,g). With increasing altitude, L/W decreased significantly (*R^2^* = 0.170, *p* = .024; Figure 5h). However, there was no significant correlation between leaf width and altitude (*p* = .559; Figure 5e). Additionally, leaf length, width, and L/W had no linear correlation with the longitude (*p* > .05; Figure 5c,f,i).

**FIGURE 6:**
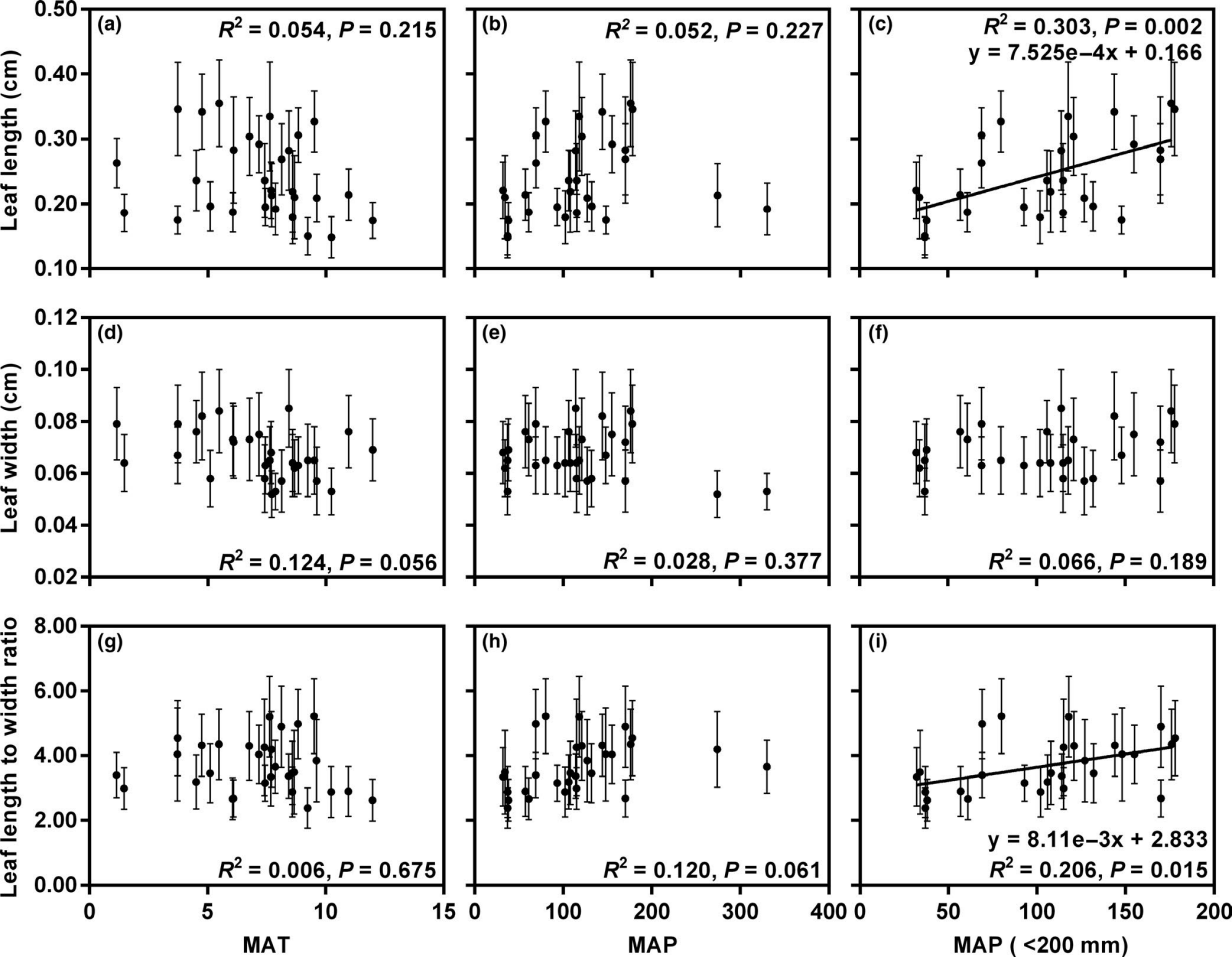
Relationships between leaf traits and climatic factors for the 30 in situ populations of *Reaumuria soongarica.* (c, f, i) The populations with MAP more than 200 mm were removed in these linear regression analyses. The value for each trait in each population is the mean ± *SD*. Units: MAT (°C); MAP (mm)

Both of the leaf length and L/W first increased and then decreased with increasing MAT and MAP (Figure 6a,b,g,h). After removing the LHT and HLG populations (MAP > 200 mm), leaf length and L/W of the remaining populations showed significant positive correlations with MAP (*R^2^* = 0.303, *p* = .002 and *R^2^* = 0.206, *p* = .015, respectively; Figure 6c,i). However, leaf width was uncorrelated with MAT or MAP (*p* > .05; Figure 6d–f).

To further elucidate the effects of the entire environment on leaf traits, stepwise regression analyses were performed using longitude, latitude, altitude, and 19 bioclimatic variables as independent factors. Results indicated that 52.74% of the variation in leaf length could be explained by the latitude (latitude was the greatest driver of leaf length variation) and precipitation of the driest quarter (bio17). The biggest factors influencing leaf width variation were the mean temperature of the coldest quarter (bio11) and precipitation of the wettest quarter (bio16), which accounted for 32.46% of the variation. In the optimal regression equation of L/W, precipitation of the driest month (bio14) was the most influential factor, accounting for 24.02% of the variation (Table 4).

**TABLE 4:** Optimal stepwise regression equation of each measured leaf trait of the 30 in situ populations of *Reaumuria soongaric*a

Leaf trait	Regression equation	*R^2^*	Adjusted *R^2^*	*F*	*p*	Coefficients					
						Factor	Unstandardized Coefficients		S.C.	*t* test	*p*
							*B*	*SE*	Beta		
Leaf length	y = −0.1349 + 0.0084x_2_ + 0.0036x_20_	0.560	0.527	17.184	.000	Constant	−0.1349	0.133		−1.015	.319
						x_2_ (latitude)	0.0084	0.003	0.423	2.471	.020
						x_20_ (bio17)	0.0036	0.002	0.397	2.316	.028
Leaf width	y = 0.0571 – 0.0017x_14_ ‐ 0.0001x_19_	0.371	0.325	7.968	.002	Constant	0.0571	0.005		10.696	.000
						x_14_ (bio11)	−0.0017	0.000	−0.508	−3.328	.003
						x_19_ (bio16)	−0.0001	0.000	−0.339	−2.219	.035
L/W	y = 3.2498 + 0.2169x_17_	0.266	0.240	10.168	.004	Constant	3.2498	0.189		17.218	.000
						x_17_ (bio14)	0.2169	0.068	0.516	3.189	.004

Note

Annotations of bioclimatic factors are shown in Table 2.

Abbreviations: L/W, leaf length to width ratio; S.C., standardized coefficients; *SE*, standard error.

### 3.4 Common garden experiment

**FIGURE 7:**
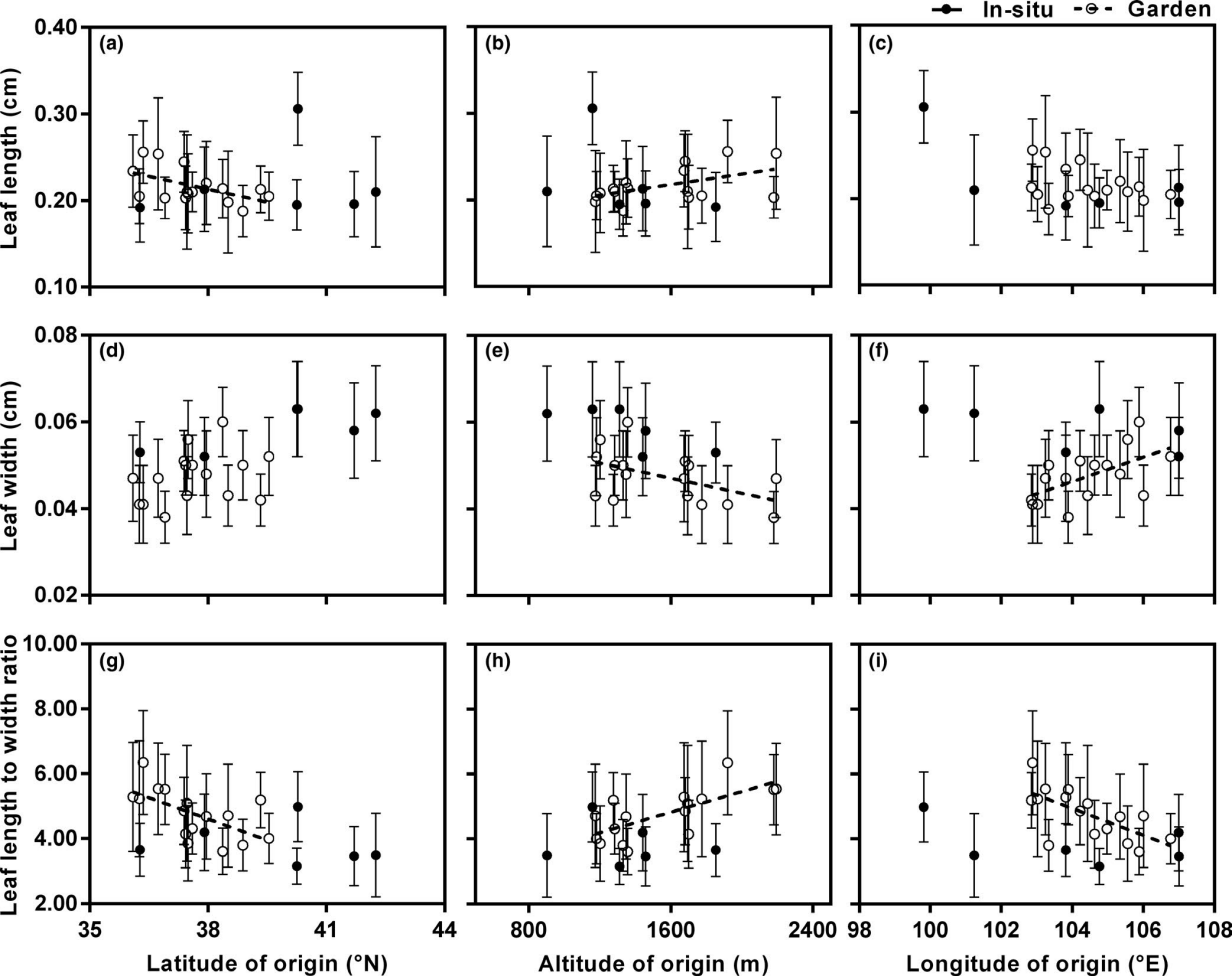
Relationships between leaf traits and geographic factors for the common garden populations and six in situ populations from the BJTD group of *Reaumuria soongarica.* (a) Leaf length with latitude (in situ populations, *R^2^* = 0.021, *p* = .786; common garden populations, *R^2^* = 0.275, *p* = .037). (b) Leaf length with altitude (in situ, *R^2^* = 0.165, *p* = .425; common garden, *R^2^* = 0.271, *p* = .039). (c) Leaf length with longitude (in situ, *R^2^* = 0.465, *p* = .136; common garden, *R^2^* = 0.131, *p* = .168). (d) Leaf width with latitude (in situ, *R^2^* = 0.579, *p* = .079; common garden, *R^2^* = 0.110, *p* = .209). (e) Leaf width with altitude (in situ, *R^2^* = 0.564, *p* = .085; common garden, *R^2^* = 0.252, *p* = .047). (f) Leaf width with longitude (in situ, *R^2^* = 0.370, *p* = .200; common garden, *R^2^* = 0.343, *p* = .017). (g) Leaf length to width ratio (L/W) with latitude (in situ, *R^2^* = 0.032, *p* = .736; common garden, *R^2^* = 0.371, *p* = .012). (h) L/W with altitude (in situ, *R^2^* = 0.017, *p* = .805; common garden, *R^2^* = 0.503, *p* = .002). (i) L/W with longitude (in situ, *R^2^* = 0.202, *p* = .371; common garden, *R^2^* = 0.446, *p* = .005). In (a) to (i), open circles and dashed fitted lines represent the garden populations, and filled circles represent the in situ populations from the BJTD group. The value for each trait in each population is the mean ± *SD*

Leaf length, leaf width, and L/W were all significantly different among the populations in the common garden experiment (*p* < .001; Figure 2b; Table 5). Compared with the wild samples from the same genetic group, BJTD, the leaf traits in the garden group showed significant differences, including leaf length (*p* = .044), leaf width (*p* < .001), and L/W (*p* < .001). Meanwhile, the CV of the leaf length in the garden group was much smaller than that in the wild BJTD group, but the CV of the leaf width in the garden group was slightly larger (Tables 3 and 5). The phenotypic differentiation in leaf width between garden populations was larger than those between wild populations in the BJTD group (Tables 3 and 5; Figure 7d).

**TABLE 5:** Statistics of measured leaf traits for *Reaumuria soongarica* in the common garden

Group	Population Code	Leaf length (cm)		Leaf width (cm)		Leaf length to width ratio	
		Mean ± *SD*	CV	Mean ± *SD*	CV	Mean ± *SD*	CV
BJTD		0.217 ± 0.045	0.208	0.048 ± 0.010	0.209	4.771 ± 1.446	0.303
	DGD	0.245 ± 0.035^a^	0.144	0.051 ± 0.007^bc^	0.143	4.856 ± 1.032^bc^	0.212
	HG	0.205 ± 0.032^cd^	0.157	0.041 ± 0.009^gh^	0.211	5.229 ± 1.776^b^	0.340
	HSW	0.256 ± 0.036^a^	0.144	0.041 ± 0.009^gh^	0.211	6.339 ± 1.604^a^	0.253
	MQ	0.188 ± 0.030^e^	0.159	0.050 ± 0.008^bcd^	0.160	3.799 ± 0.799^ef^	0.210
	XGG	0.203 ± 0.024^cd^	0.120	0.038 ± 0.006^h^	0.146	5.515 ± 1.086^ab^	0.197
	RSS	0.254 ± 0.065^a^	0.256	0.047 ± 0.009^def^	0.190	5.529 ± 1.405^ab^	0.254
	JZ	0.234 ± 0.042^ab^	0.179	0.047 ± 0.010^def^	0.205	5.287 ± 1.681^b^	0.318
	QYS	0.208 ± 0.046^cd^	0.220	0.056 ± 0.009^ab^	0.170	3.854 ± 1.162^ef^	0.302
	SSC	0.210 ± 0.066^cd^	0.315	0.043 ± 0.009^efg^	0.219	5.084 ± 1.789^bc^	0.352
	YWQ	0.203 ± 0.037^cd^	0.182	0.050 ± 0.007^bcd^	0.149	4.147 ± 1.044^def^	0.252
	SRT	0.213 ± 0.027^bc^	0.128	0.042 ± 0.006^gh^	0.135	5.188 ± 0.851^b^	0.164
	THCY	0.210 ± 0.023^cd^	0.109	0.050 ± 0.007^cd^	0.151	4.311 ± 0.785^cde^	0.182
	WD	0.205 ± 0.028^cd^	0.135	0.052 ± 0.009^bc^	0.171	4.001 ± 0.778^def^	0.194
	YH	0.220 ± 0.048^bc^	0.217	0.048 ± 0.010^cde^	0.202	4.685 ± 1.313^cd^	0.280
	SGK	0.214 ± 0.034^bc^	0.158	0.060 ± 0.008^a^	0.129	3.605 ± 0.716^f^	0.199
	SMY	0.198 ± 0.059^de^	0.298	0.043 ± 0.007^fg^	0.160	4.707 ± 1.589 ^cd^	0.338
*R. trigyna*		0.720 ± 0.210	0.291	0.071 ± 0.011	0.156	10.222 ± 3.062	0.300

Note

In each column, the mean of populations with different lowercase letters (*p* < .05) are significantly different.

Abbreviations: CV, coefficient of variation; *SD*, standard deviation.

**FIGURE 8:**
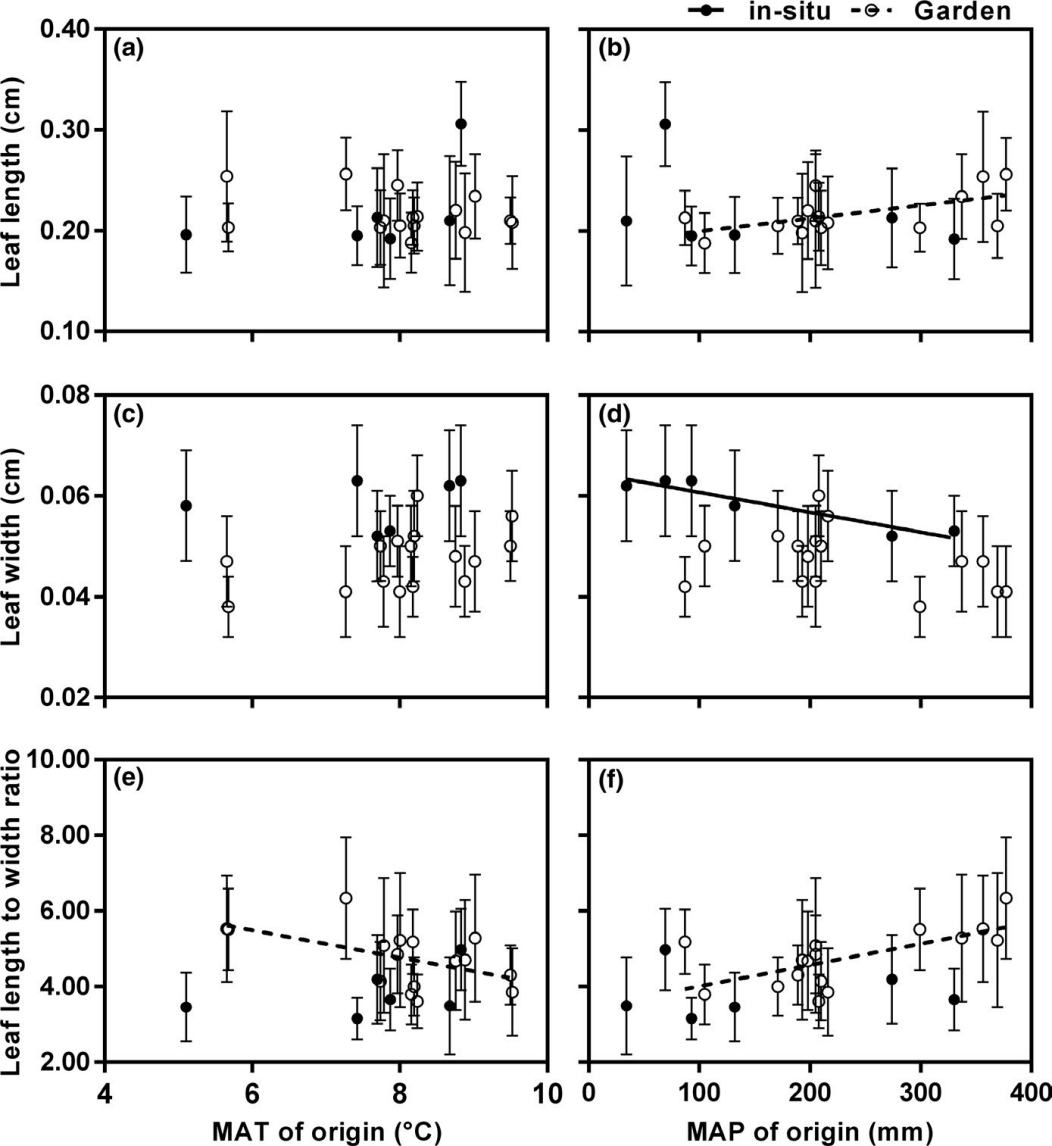
Relationships between leaf traits and climatic factors for the common garden populations and six in situ populations from the BJTD group of *Reaumuria soongarica.* (a) Leaf length with MAT (in situ populations, *R^2^* = 0.266, *p* = .295; common garden populations, *R^2^* = 0.103, *p* = .226). (b) Leaf length with MAP (in situ, *R^2^* = 0.142, *p* = .461; common garden, *R^2^* = 0.327, *p* = .021). (c) Leaf width with MAT (in situ, *R^2^* = 0.052, *p* = .665; common garden, *R^2^* = 0.198, *p* = .084). (d) Leaf width with MAP (in situ, *R^2^* = 0.886, *p* = .005; common garden, *R^2^* = 0.125, *p* = .180). (e) Leaf length to width ratio (L/W) with MAT (in situ, *R^2^* = 0.219, *p* = .350; common garden, *R^2^* = 0.280, *p* = .036). (f) L/W with MAP (in situ, *R^2^* = 0.0001, *p* = .983; common garden, *R^2^* = 0.434, *p* = .006). In (a) to (f), open circles and dashed fitted lines represent the garden populations; filled circles and solid fitted lines represent the in situ populations from the BJTD group. The value for each trait in each population is the mean ± *SD*

Both the leaf length and L/W of the garden populations were positively correlated with the altitude of origin (Figure 7b,h) and MAP of origin (Figure 8b,f), but negatively correlated with the latitude of origin (Figure 7a,g). The L/W of the garden populations was negatively correlated with the longitude of origin (Figure 7i) and MAT of origin (Figure 8e). Meanwhile, the leaf width of the garden populations increased significantly with decreasing altitude of origin and increasing longitude of origin (Figure 7e,f). There was a significant negative correlation between MAP and leaf width in the wild populations from BJTD (*p* = .005), but this pattern disappeared in the garden populations (*p* = .180; Figure 8d). Except for the relationship between leaf width and MAP, these three leaf characteristics in wild populations from BJTD were unrelated to any of the environmental factors analyzed (Figures 7 and 8).

**FIGURE 9:**
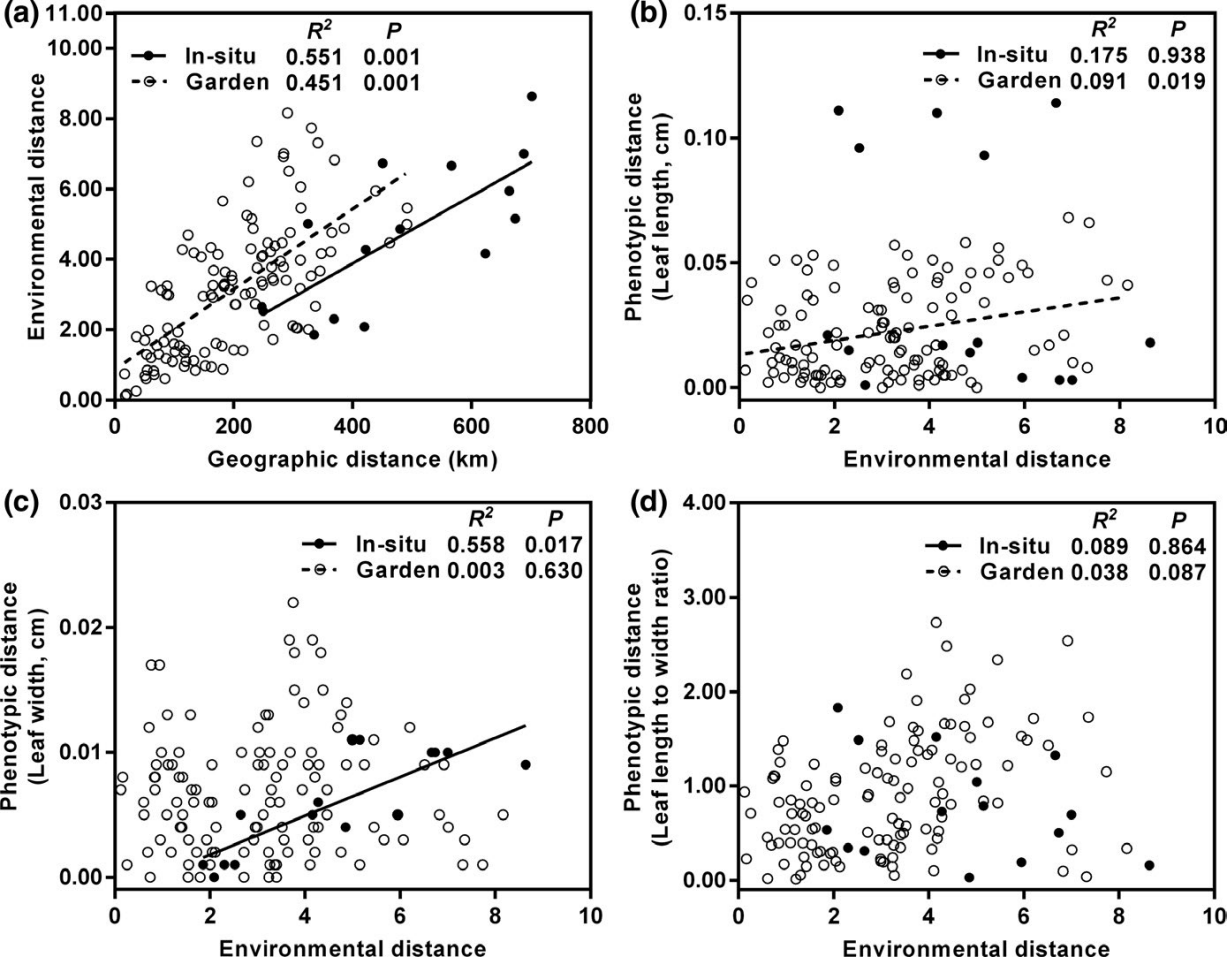
Partial Mantel tests between the phenotypic distance of leaf traits and environmental distance for the common garden populations and six in situ populations from the BJTD group of *Reaumuria soongarica.* (a) Mantel tests between environmental distance and geographic distance among the common garden populations and among six in situ populations from the BJTD group. (b–d) Partial mantel tests between the environmental distance and phenotypic distance of leaf length, leaf width, and leaf length to width ratio. In each panel, *R^2^* and *P* value were estimated by Mantel test or partial Mantel test. Fitted slopes were estimated by linear regression analyses

Partial Mantel tests revealed a significant relationship between the phenotypic distance of leaf length in the garden populations and environmental distance between their original sites (*p* = .019), but there was no such correlation for the wild populations from BJTD (*p* = .938; Figure 9b). In contrast, the phenotypic distance of leaf width between in situ populations from BJTD was positively related to environmental distance (*p* = .017), but this correlation was not seen in the garden populations (*p* = .63; Figure 9c). There was no significant correlation between the phenotypic distance of L/W and environmental distance in either the wild or garden populations (*p* > .05; Figure 9d). In addition, the range of leaf width distance and L/W distance of the garden populations was larger than that of the wild populations from BJTD (Figure 9c,d).

## 4 DISCUSSION

Very low precipitation and high temperatures characterize the growing season in ACA, and there are substantial environmental differences between different regions (Chen et al., 2011; Hu et al., 2014). Although these environmental conditions are unfavorable for plant growth, *R. soongarica* is widespread in these areas (Liu et al., 1982; Shi et al., 2013). According to our data, the leaf area of this species was less than 0.15 cm^2^, which was smaller than that of most species in the world (Diaz et al., 2016; Wright et al., 2017). Small leaves can maintain their leaf temperature within a favorable range in hot and dry environments in ACA through rapid sensible heat exchange of their thinner boundary layer (Leigh et al., 2017; Parkhurst & Loucks, 1972; Wright et al., 2017), which is essential for photosynthesis and respiration of plants (Berry & Bjorkman, 1980; Gates, 1965). Additionally, the leaf size of this shrub varied greatly among the 30 wild populations sampled, indicating that this trait has high plasticity or adaptability under different environmental conditions. Undoubtedly, these characteristics of *R. soongarica* have contributed to its successful distribution in a wide range of arid environments. Research into the relationship between leaf size and the environment is, therefore, important to improve our understanding of how *R. soongarica* adapts and responds to heterogeneous arid environments across ACA.

### 4.1 Phenotypic differentiation of leaf size among genetic lineages

Based on molecular evidence, *R. soongarica* can be divided into three genetic lineages (Shi et al., 2020; Yin et al., 2015), which occupy different niches and geographic areas. The northern lineage might have originated as a hybrid between the eastern and western lineages and is in the early stage of ecological speciation (Shi et al., 2020). In this study, we found that leaf length, leaf width, and L/W of the northern lineage were significantly larger than those of the other two lineages (Figure 3b–d). Meanwhile, there were obvious environmental differences between the three genetic lineages (Figure 4a; Table 2). Similarly, the needle length of *Pinus densata* (a homoploid hybrid species) is intermediate between that of its parental species, *P. tabuliformis,* and *P. yunnanensis*, and the environments occupied by these three species are also different (Xing et al., 2014). Li et al. (2014) suggested that the divergences of leaf area and ecological niche between *Aquilegia japonica* and *A. oxysepala* were associated with their ecological speciation. In fact, the morphological differentiation of plant traits, caused by natural divergent selection in different niches, has been observed in many cases of ecological speciation and might be a driving force for ecological speciation (Bradshaw & Schemske, 2003; Li et al., 2014; Medrano, Castellanos, & Herrera, 2006; Minnaar, de Jager, & Anderson, 2019; Xing et al., 2014). However, the phenotypic differentiations of leaf traits cannot directly lead to reproductive isolation. Thus, we argue that the observed phenotypic differentiation of leaf size among genetic lineages of *R. soongarica* may be a by‐product of the ecological speciation, which may be caused by long‐term adaptation to different environments.

Although the leaf length of *R. soongarica* varied considerably among three genetic lineages, the ranges partially overlapped (Figure 3b). However, the leaf length of *R. soongarica* did not exceed that of *R. trigyna* and *R. kaschgarica*, and there was almost no overlap in the range of leaf length between *R. soongarica* and the other two species. This implies that the leaf length of *R. soongarica* changes within a certain range, no matter how stressful or favorable the surrounding conditions. In fact, in any given species of plant, the size of an organ is maintained within a restrictive range, even across different environments, and is genetically determined (Powell & Lenhard, 2012). Hence, based on our data on leaf length, we speculate that the three genetic lineages of *R. soongarica* are not yet completely differentiated.

Compared to leaf size, the phenotypic differentiations of floral traits are more closely related to the evolution of reproductive isolation in plants (Bradshaw & Schemske, 2003; Hodges, Whittall, Fulton, & Yang, 2002). For example, reproductive isolation between *Mimulus lewisii* and *M. cardinalis* is directly caused by different flower colors attracting different pollinators (Bradshaw & Schemske, 2003). In *R. soongarica*, the flowering time of the northern lineage was intermediate between that of the western and eastern lineages, which did not overlap with themselves (X. Fan & X.‐F. Ma, unpublished data). These differences could gradually have induced the reproductive isolation between lineages. Overall, these observed differentiations in leaf size and flowering time between genetic lineages of *R. soongarica* support the opinion that the northern lineage may be an incipient species arising from ecological speciation (Shi et al., 2020). To further confirm that these morphological differentiations in *R. soongarica* are due to long‐term adaptation to different environments, future work should involve planting the populations from all lineages in a common garden in each region where the three lineages are located, to observe whether these phenotypic differentiations persist.

### 4.2 Unique relationships between leaf size and environmental factors in ACA

Recently, Wright et al. (2017) characterized the global latitudinal trend of leaf size by using leaf data from 7,670 plant species at 682 sites worldwide and demonstrated that latitude could explain 28% of leaf size variation globally. In their study, however, only three very close sites were sampled from ACA, while fewer than ten sites were located in arid regions, including the Sahara Desert, ACA, and central Australia. Thus, more research is needed to describe the relationships between leaf size and environmental factors in arid regions. Our results showed opposite trends in wild *R. soongarica* in ACA; leaf length, leaf width, and L/W significantly increased with increasing latitude (Figure 5a,d,g). Furthermore, linear regression analyses suggested that latitude could explain 47.27% of leaf length variation and 24.64% of leaf width variation in this species. Even when all environmental factors were considered, latitude was still the major factor affecting the leaf length variation in *R. soongarica* (Table 4).

This latitudinal trend of leaf size in *R. soongarica* supplied a new evidence for the prediction on maximum leaf sizes in arid regions (Wright et al., 2017). In their prediction, maximum leaf sizes of plants in arid regions are smaller than those of plants from adjacent higher latitude areas. However, to date, it is far from clear how leaf size varies in response to the environmental heterogeneity in a local arid region, such as ACA. With a focus on temperature and precipitation, there are three reasons that might, to some extent, explain this unique trend of leaf size in *R. soongarica*. First, the sampled sites with lower precipitation were concentrated in lower latitudes (Table 1). Second, the altitudes of the sites we collected increased with decreasing latitude (Table 1), indicating that the sites in lower latitudes have higher solar radiation, resulting in higher transpiration (Korner, 2007). Third, during the early growing season, the soil moisture in higher latitudes (the Gurbantunggut Desert) is replenished by snowmelt (Fan, Tang, Wu, Ma, & Li, 2014). Therefore, *R. soongarica* may face more serious drought stress at relatively lower latitudes and could have adapted to avoid heat damage by producing smaller leaves.

In addition, our results showed that the leaf length of wild *R. soongarica* first increased and then decreased with increasing MAT and MAP (Figure 6a,b). For example, the populations WXBS, MF, and PYLM had the shortest leaf length and approximately the lowest MAP and the highest MAT, while the populations with the longest leaf length, WCC, BEJ, and QKET, had medium levels of MAP and MAT (Tables 1 and 3). These nonlinear relationships described above indicate that any single climatic factor cannot completely explain the variation in leaf size, and the combined effects of climatic factors on leaf size are complicated (Parkhurst & Loucks, 1972; Sun et al., 2016; Wright et al., 2017). In other words, different leaf sizes under different combinations of temperature and precipitation are used to maintain the leaf temperature within a normal range while maximizing the benefit for plant growth.

The spatial relationships between leaf size and environmental factors are commonly used to predict the impacts of climate change on ecosystem functioning through space‐for‐time substitution (Bjorkman et al., 2018; Myers‐Smith et al., 2019). In past decades, the MAT and MAP in ACA have generally increased, and these changes differed regionally (Chen et al., 2011; Hu et al., 2014). Combining our results with the detailed climate data, the response of leaf size of *R. soongarica* to climate change in different regions could be predicted. Due to sparse vegetation and few plant species in ACA, the variations in leaf size of *R. soongarica* can fully reflect the consequences of climate change on the functioning of desert ecosystems dominated by this shrub. To get more accurate predictions about the ecological consequences of climate change in ACA, more environmental factors such as wind speed (Yin, Qian, et al., 2016), rainfall interval (Zhang et al., 2018), soil nutrients (McDonald et al., 2003), and UV‐B (Sun et al., 2016) should also be considered as these also strongly affect the functional traits of desert plants. It is better to use the variations in plant traits on a large time scale to verify the accuracy of these predictions (Bjorkman et al., 2018; Hudson et al., 2011), but such data are scarce for ACA.

### 4.3 Stability and local adaptation of leaf width in *R. soongarica*

Leaf size can change through several combinations of leaf width variations and leaf length variations among and within lineages (McDonald et al., 2003). Previous studies have found that leaf widths of plant species are usually negatively correlated with altitude (Guo, Lin, Chen, & Yang, 2018; Hovenden & Vander Schoor, 2004; Sun et al., 2016). In this study, leaf length and L/W of wild *R. soongarica* significantly decreased with increasing altitude (Figure 5b,h), while leaf width was unrelated to altitude (Figure 5e). Like altitude, the MAP and MAT also had less of an effect on leaf width than on leaf length of this shrub (Figure 6). Taken together, these results suggest that leaf width is more stable than leaf length in *R. soongarica*, and this shrub prioritizes changing leaf length to adjust leaf size to cope with environmental change. Additionally, we found that the leaf widths between the three sister species in genus *Reaumuria* endemic to ACA were also relatively stable (Table 3). Leaf boundary layer thickness affects the rate of heat exchange between leaves and the surrounding air and is mainly determined by leaf width (Leigh et al., 2017; Parkhurst & Loucks, 1972). Thus, the small and relatively stable leaf width of *R. soongarica* means this shrub can always maintain an appropriate rate of heat exchange and therefore avoid potential heat or frost damage in the harsh environments found in ACA (which experiences extremely low precipitation and large diurnal temperature variations).

The high genetic differentiation between the three lineages of *R. soongarica* (Shi et al., 2020; Yin et al., 2015) would complicate our interpretations of the effects of environmental and genetic factors on the variations in leaf traits. Thus, we only used the populations from the eastern lineage to conduct the common garden experiment. In this study, the leaf width of *R. soongarica* varied considerably among populations in the common garden (Table 5). Compared to the wild populations from the same genetic group, the phenotypic differentiations in leaf width between garden populations were greater, and the range of leaf width distance was also larger (Tables 3 and 5; Figures 7d and 9c). Without environmental differences, these larger variations in leaf width in the common garden must be caused by genetic factors, that is, the result of different populations adapting to the different original environments. The strong relationship between leaf width in garden populations and the longitudes of the origins also supports this opinion (Figure 7f). However, unlike the known adaptive traits in other species (Cordell et al., 1998; Zhu et al., 2012), the strong correlation between leaf width distance and environmental distance in wild *R. soongarica* populations (BJTD) disappeared in the common garden (Figure 9c). Therefore, we believe that the smaller leaf width variation among wild *R. soongarica* populations distributed along the environmental gradient is not only caused by local adaptation, but also affected by phenotypic plasticity. This is also seen in *Poa hiemata*; altitudinal trends in leaf length and plant circumference in this species are affected by both genetic and environmental factors (Byars et al., 2007). Unfortunately, we could not give a specific percentage for the contribution of local adaptation and phenotypic plasticity to the leaf width variations, because the populations used in the common garden experiment in this study did not correspond to the wild populations from the same genetic group.

## 5 CONCLUSIONS

*Reaumuria soongarica* is an excellent model to understand how desert plants endemic to ACA adapt and respond to environmental change. Our study shows that this desert shrub has a unique latitudinal gradient of leaf size. Based on the field sampling and common garden experiment, we found that the leaf width of *R. soongarica* is more stable than leaf length, which suggests that this species prioritizes changing leaf length to adjust leaf size to cope with environmental change. Furthermore, the northern hybrid lineage prevailed over its two parental lineages in all productivity‐related traits investigated, providing phenotypic evidence for ecological speciation within this shrub. Of course, to dissect the specific contributions of plasticity and genetic differentiation on phenotypic variations of *R. soongarica*, and to further study the impact of these variations on ecological speciation, more functional traits, including flowering time, should be analyzed in different transplant experiments.

## CONFLICT OF INTEREST

The authors have no conflict of interest to declare.

## AUTHOR CONTRIBUTIONS

**Xingke Fan:** Conceptualization (supporting); Data curation (equal); Formal analysis (lead); Investigation (equal); Methodology (equal); Software (lead); Visualization (lead); Writing‐original draft (lead); Writing‐review & editing (equal). **Xia Yan:** Conceptualization (equal); Formal analysis (supporting); Methodology (equal); Project administration (equal); Writing‐original draft (supporting); Writing‐review & editing (equal). **Chaoju Qian:** Data curation (equal); Funding acquisition (supporting); Investigation (supporting); Project administration (equal); Resources (equal); Writing‐review & editing (equal). **Daoura Goudia Bachir:** Data curation (supporting); Investigation (equal); Resources (supporting); Writing‐review & editing (equal). **Xiaoyue Yin:** Data curation (equal); Formal analysis (supporting); Investigation (equal); Methodology (supporting); Resources (equal); Writing‐review & editing (supporting). **Peipei Sun:** Data curation (equal); Formal analysis (supporting); Investigation (equal); Methodology (supporting); Resources (supporting); Writing‐review & editing (supporting). **Xiao‐Fei Ma:** Conceptualization (lead); Formal analysis (supporting); Funding acquisition (lead); Methodology (equal); Project administration (lead); Supervision (lead); Writing‐original draft (supporting); Writing‐review & editing (lead).

## Data Availability

Morphological and climate data used in this study are deposited in the Dryad data repository (https://doi.org/10.5061/dryad.dv41ns1vr).
